# Development of a Real-Time Cell Analysis (RTCA) Method as a Fast and Accurate Method for Detecting Infectious Particles of the Adapted Strain of Hepatitis A Virus

**DOI:** 10.3389/fcimb.2018.00335

**Published:** 2018-09-25

**Authors:** Samuel Lebourgeois, Audrey Fraisse, Catherine Hennechart-Collette, Laurent Guillier, Sylvie Perelle, Sandra Martin-Latil

**Affiliations:** Laboratory for Food Safety, Université Paris Est, ANSES, Maisons-Alfort, France

**Keywords:** hepatitis A virus, infectious virus, quantification, real-time cell analysis (RTCA) assay, cell impedance

## Abstract

Hepatitis A virus (HAV) is one of the most common agents causing acute liver disease worldwide. HAV has been increasingly reported as the cause of foodborne disease outbreaks. The standard method currently available for detection of the genome of HAV in vulnerable foodstuffs is by RT-qPCR (ISO 15216). Despite its usefulness in the investigation of foodborne viruses, the use of RT-qPCR in food virology has been shown to overestimate the quantity of infectious virus or to highly underestimate the effect of the treatment on virus inactivation. The gold standard methods currently used for evaluating the efficacy of inactivation treatments on the adapted strain of HAV (HM175/18f) are either the plaque assay or the end-point dilution assay (TCID_50_). However, both assays are labor-intensive and time-consuming. The aim of this study was to evaluate the use of the xCELLigence real-time cell analysis (RTCA) system for detecting the infectivity of the adapted strain of HAV. Kinetics of cell impedance showed that HAV induced a decrease in cell index (CI) correlated with the onset of HAV-induced cell death. In addition, the time to which the HAV-induced CI drop occurred was dependent on the viral concentration. An inverse linear relation could be established over a range of 5 log_10_ between the concentration of HAV and the time to reach 50% of CI decrease (TCI_50_), showing that the RTCA assay could be used as a titration method for HAV. In addition, the RTCA-based assay could be performed in less than 6 days instead of 12 to 14 days with the gold standard methods. Therefore, the RTCA-based titration method is a powerful and suitable tool for high-throughput screening of anti-viral treatments. Its usefulness in HAV inactivation studies will improve the assessment of viral risk in food virology, as controlling transmission of viruses through their removal from foodstuffs is also an important challenge in reducing the burden of viral foodborne illnesses.

## Introduction

Hepatitis A virus (HAV) is a nonenveloped virus with a single-stranded, positive-sense RNA genome of the genus Hepatovirus in the *Picornaviridae* family (Vaughan et al., 2014). HAV is primarily transmitted to humans through the fecal–oral route. The disease, acute and generally self-limiting, affects the liver and is characterized by fever, diarrhea, and jaundice. Severity of disease is strongly associated with age, with older children and adults often experiencing symptomatic disease (Koff, 1992; Mohd Hanafiah et al., 2011). The incidence rate of HAV infection is closely related to socioeconomic factors that affect the quality of sanitation and access to safe drinking water. In the last two decades, improved hygiene has led to a change in its epidemiology. The outcome is an increase in HAV outbreaks in developed countries, where young individuals and adults are susceptible, favoring the occurrence of hepatitis A outbreaks caused by imported food contaminated with the HAV (Gallot et al., 2011; Carvalho et al., 2012; Severi et al., 2015).

To ensure the safety of food products, it is important to develop sensitive, rapid and reliable methods for the detection of HAV to check the absence of viral agents and to assess the efficacy of technological treatments implemented in food industries for virus removal. The ISO 15216 standard (ISO, 2017) is based on a final detection of the viral genome using real-time reverse transcriptase PCR (RT-qPCR). In food virology, the use of RT-qPCR has been shown to overestimate the quantity of infectious virus or to highly underestimate the effect of the treatment on virus inactivation (Simonet and Gantzer, 2006; de Roda Husman et al., 2009; Fraisse et al., 2011). Therefore, finding an effective method for detecting infectious viral particles is currently crucial for improving the assessment of viral risk. The cell culture system remains the gold standard method for detecting infectious viral particles. Because the wild-type of HAV is not routinely cultivable *in vitro*, the adapted strains are frequently used to assess the virucidal efficiencies of inactivation treatments. However, both methods currently used as titration methods of HAV adapted strains (i.e., the viral plaque and median tissue culture infective dose (TCID_50_) assays) are endpoint assay, time-consuming and labor intensive (Anderson, 1987; Cromeans et al., 1987).

Recently, an innovative Real-Time Cell Analysis (RTCA) technology based on cellular impedance measurements has been used to monitor the cytopathic effects (CPE) induced by several viruses belonging to different families (e.g., flavivirus, influenza A virus (H1N1), HEV71, chikungunya virus) as well as for evaluating neutralizing antibodies and/or antiviral drugs (Fang et al., 2011; Tian et al., 2012; Teng et al., 2013; Marlina et al., 2015). The xCELLigence system (ACEA Biosciences) measures changes in electrical impedance (CI, cell index value) of cell monolayers using specialized cell culture microplates containing micro-electrodes. It allows dynamic real-time, label-free, and non-invasive analysis of cellular events such as cell adhesion, spreading, proliferation (growth), cell viability, cell death, and detachment, thus creating a picture of cell function during viral infection.

In this study, the xCELLigence real-time cell analysis (RTCA) system was evaluated for dynamic monitoring of cytopathic effects induced by an adapted strain of HAV (HM175/18f) on FRhK-4 (fetal rhesus monkey kidney) cells. The feasibility and efficiency of this new technology as a titration method for infectious HAV was further investigated by evaluating the impact of cell culture conditions.

## Materials and methods

### Cell line and virus stocks

The FRhK-4 (fetal rhesus monkey kidney) cell line was purchased from the American Type Culture Collection (ATCC) (ATCC® CRL-1688™) (LGC standards SARL, Illkirch, France). These epithelial cells were cultured in Dulbecco's modified Eagle's medium (DMEM, Gibco™) supplemented with non-essential amino acids (NEAA, Gibco™) and 10% of heat-inactivated fetal bovine serum (FBS, Gibco™) (Thermo Fisher Scientific, Waltham, MA, 209 USA). Cells were maintained at 37°C in a humidified atmosphere containing 95% air and 5% CO_2_.

The cell culture-adapted HM175-18f strain of HAV was obtained from ATCC (VR-1402). This clone replicates rapidly and has cytopathic effects in cell culture (Kulka et al., 2003). HAV stock was produced by propagation in FRhK-4 cells (ATCC, CRL-1688) and titrated by lysis plaque assay (Deboosere et al., 2004). Results were obtained 12 days after HAV infection and expressed in plaque-forming units/ml (PFU/ml). HAV stock titer was 5 × 10^6^ PFU/ml. The supernatant was aliquoted for storage at −80°C.

### Impedance measurement with the xCELLigence DP system

The xCELLigence DP system [Ozyme (Montigny Le Bretonneux, France) and ACEA Biosciences (San Diego, CA, USA)] is a Real-Time Cell Analyzer (RTCA) based on the assessment of cell-impedance variations. This RTCA system comprises four main components: the RTCA DP Station with three independent E16-well plate platforms placed inside a tissue-culture incubator; the RTCA sensor Analyzer for sending and receiving the electronic cellular signals; the RTCA computer (Control Unit) with integrated software (RTCA Software 2.0) to acquire and display data in real time, and the disposable E-Plates 16. The E-plate is a standard 16-well plate with glass bottom coated with gold microelectrodes covering approximately 75% of the well area.

Measured electrical impedance is translated as a dimensionless parameter, the Cell Index (CI). When cells are not present or adhered, CI value is zero. When there are no cells on the electrode surface, the impedance describes only the background. In contrast, CI values increase progressively and proportionally as cells become attached to the electrodes. The CI variations are displayed in a real-time plot by the software. The data generated with the integrated software were exported to Excel and images were digitally recorded with Adobe Illustrator for further processing.

### Monitoring cell growth using the xCELLigence DP system

The growth curves of FRhK-4 cells (adhesion, proliferation and stationary phase) were established according to the cellular density at seeding using cell impedance measurements with the xCELLigence DP system (ACEA Biosciences). First, 100 μl of cell culture medium (DMEM supplemented with 10% FBS) was added to each well of E-plate 16. The E-plate 16 was then connected to the system to check for proper electrical contacts and to obtain background impedance readings in the absence of cells.

Meanwhile, the FRhK-4 cells were resuspended in the appropriate cell culture medium, adjusted to 800,000 cells/ml and serially two-fold diluted down to 6,250 cells/ml. Then, 100 μl of every cellular density was added to the wells containing 100 μl of culture medium in order to determine the optimum cell concentration. After 30 min incubation at room temperature, the E-plate 16 was placed onto the RTCA SP Station located inside the incubator (5% CO_2_; at 37°C) for continuous impedance recording. CI values were measured every minute for 4 h and then every 15 min for a period of up to 250 h.

### Viral infection assays

FRhK-4 cells were seeded onto either E-plate or 96-well plate at a density of 10,000 cells/well. At 2 days post-seeding, cells were infected with HAV using one of the three protocols depending on the concentration of FBS in the medium.

Cells were washed once with serum-free medium (Protocols 1 and 2) or medium supplemented with 2% of FBS (Protocol 3) and 30 min later infected with 100 μl per well of HAV suspension diluted in serum-free medium for Protocols 1 and 2 or in 2% FBS-medium for Protocol 3.

**Figure 1:**
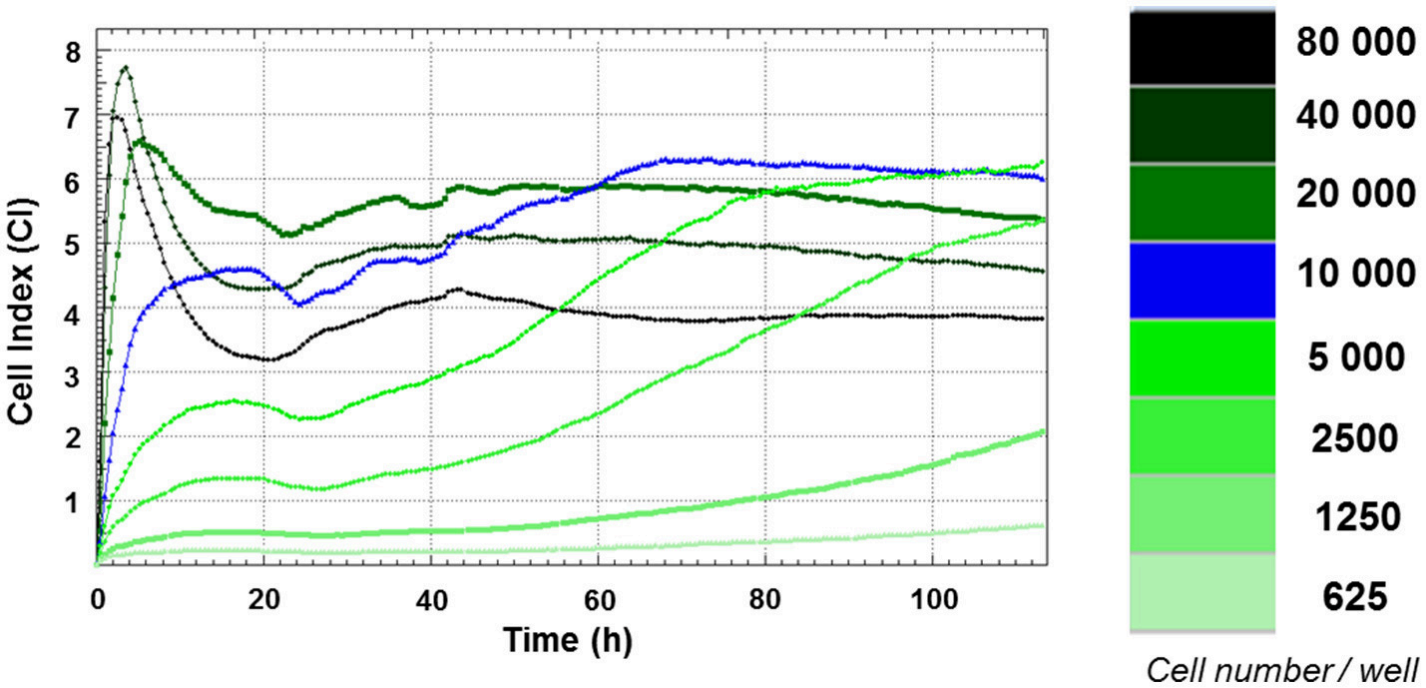
Growth curves of FRhK-4 cells established using cell impedance measurements according to the cellular density at seeding. FRhK-4 cells were seeded in E-plate 96 at different cellular densities, from 625 to 80,000 cells per well, and Cell Index (CI) values were recorded with the DP xCELLigence system to obtain the ideal cell number and to determine the most suitable time point for virus infection.

After virus adsorption for 2 h at 37°C, the viral inoculum was kept and 100 μl per well of culture medium were added to infected cells according to the protocol used: serum-free medium for Protocol 1, medium supplemented with 4% FBS or 2% FBS for Protocol 2 and Protocol 3 respectively (Figure 1).

Time zero post-infection (p.i.) corresponds to the time at which virus inoculation was performed.

### Cell viability assay

Cell viability was measured using the Cell Counting Kit-8 (CKK-8; DOJINDO) according to the manufacturer's instructions. This colorimetric assay is based on the extracellular reduction of the tetrazolium salt WST-8 (2-(2-methoxy-4-nitrophenyl)-3-(4-nitrophenyl)-5-(2,4-disulfophenyl)-2H-tetrazolium, monosodium salt) by cellular dehydrogenases of viable cells in a highly water-soluble formazan dye in the presence of an electron mediator.

Briefly, FRhK-4 cells were grown in a 96-well plate for 2 days before being infected with HAV. At the end of the time course of infection, 10 μl of the WST-8 reagent was added to each well and incubated for 3 h at 37°C in a CO_2_ incubator. To measure the absorbance later, 10 μl of 0.1 M hydrochloric acid (HCl) was added to each well and the covered plate was stored at room temperature with protection from light. No absorbance change was observed for at least 24 h.

The optical density (OD) was measured at 450 nm with the NanoDrop ND-1000 (Thermo Fisher Scientific).

The percentage of cell viability was calculated using the following formula: [OD(HAV-infected cells)—OD(blank)/(OD(mock-infected cells)—OD(blank)] × 100.

### Statistical analysis

For all statistical analyses, we used the Statgraphics Centurion XVI software.

A two-way analysis of variance (ANOVA) was performed on CI_min_ and CI_max_ values to assess (1) the effect of HAV and (2) the effect of the protocol used (i.e., cell culture medium). The result of the ANOVA is a *p* value associated with the hypothesis that the mean recovery rates of all groups were the same.

Because both CI_min_ and CI_max_ values were statistically different according to the infection protocol used (ANOVA, *p* < 0.01), a multiple comparison procedure was applied to determine which mean CI values were different. Given that there are three group means, there are also three pairs to compare. Instead of ordinary *t*-tests, Fisher's least-significant-differences (LSD) procedure was applied. The alpha-value applies to each comparison, so the chance of finding a false-positive significant difference increases with the number of comparisons. Graphs plotting the mean and its standard error for each group illustrate the multiple comparison procedure. When confidence intervals of means do not overlap, the difference between two groups of a factor is significant.

## Results

### Impedance-based growth curves of FRhK-4 cells according to the number of cells at seeding

Growth curves of FRhK-4 cells, based on cellular impedance measurement, were established according to the number of cells seeded in E-plates (from 625 to 80,000 cells per well) to get the optimal cellular density to infect cells in a sub-confluence state with HAV at 2 days post-seeding. Cell index values were recorded in real-time for a period of up to 115 h by the x-CELLigence system, and proliferation profiles of FRhK-4 cells are displayed in Figure 1.

For cellular densities higher than 2,500 cells per well, CI values increased rapidly. As expected, they increased faster when a higher number of cells were seeded. This increase corresponded to cell adhesion and occurred between 2 and 10 h following the seeding. While the CI remained stable and/or decreased slightly for cellular densities from 2,500 to 10,000 cells per well for up to 24 h, there was a drop in CI values for cellular densities from 20,000 to 80,000 cells per well. This phase was correlated with the spreading of FRhK-4 cells.

After adhesion and spreading of cells, CI values increased during the cell growth stage to reach the stationary phase. The cell growth stage lasted about 20 h for the highest cell densities (from 20,000 to 80,000 cells per well) and increased as the cell densities at seeding decreased. Indeed, the stationary phase was reached at a later time point for cell densities of 10,000 cells/well (66 h post-seeding) and 5,000 cells/well (90 h post-seeding). Regarding the cellular density of 10,000 cells/well, the cell growth phase began from 24 h to reach the stationary phase at 66 h indicating the potential time point for the infection at between 2 and 3 days post-seeding when cells are in a sub-confluence state.

As a whole, the impedance-based profiles showed the three phases of the cell growth curve (cell adhesion and spreading, the cell growth phase and the stationary phase), and a cellular density of 1 × 10^4^ cells/well was selected as the optimum number for cell seeding, while the optimal time point for viral infection was defined at 48 h after cell seeding.

### Monitoring of the HAV-induced cytopathic effect in FRhK-4 cells using real-time cell analysis (RTCA)

**Figure 2:**
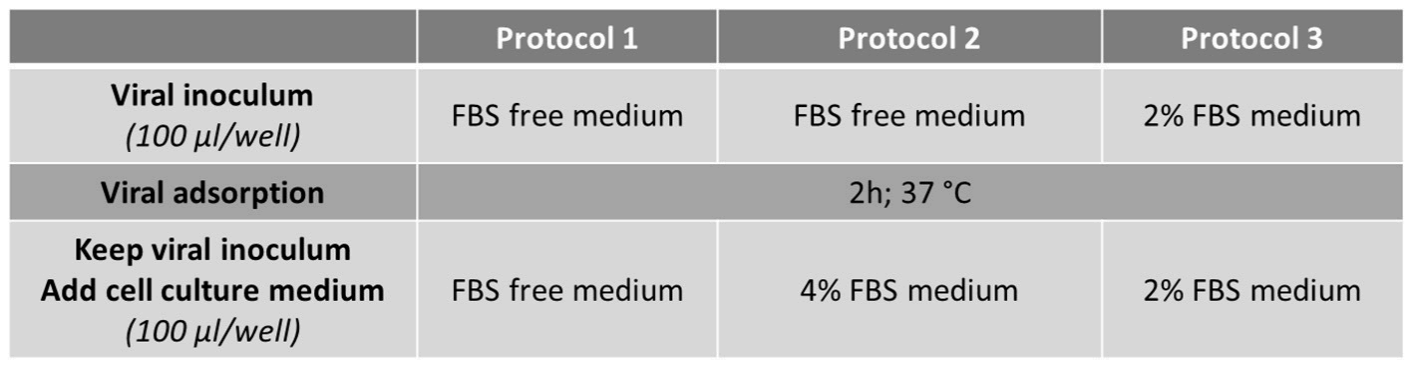
Flowchart of the three infection protocols tested for the monitoring of HAV-induced cytopathic effect in FRhK-4 cells by using Real-Time Cell Analysis (RTCA).

To evaluate the RTCA-based assay as a method for measuring the cytopathic effect of HAV, the cellular impedance of FRhK-4 cells infected or not with HAV was measured in real-time using the xCELLigence system. Viral infection was performed using one of the three protocols whose difference is based on the percentage of FBS during the viral adsorption step and the full duration of viral infection (Figure 2).

**Figure 3:**
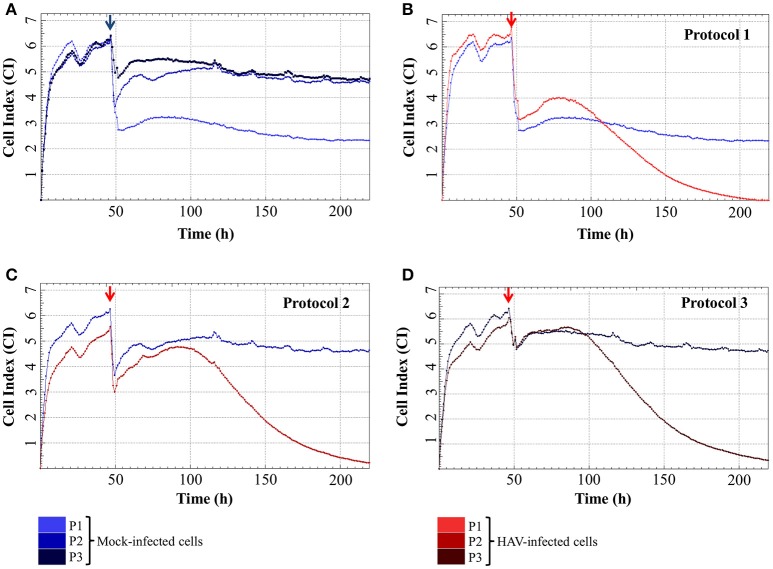
Real-time impedance analysis of mock- and HAV-infected FRhK-4 cells according to the infection protocol. **(A)** Cell Index values of mock-infected cells according to the infection protocol used. **(B–D)** Cellular impedance in mock (blue) and HAV-infected cells (red) using Protocol 1 **(B)**, Protocol 2 **(C)**, and Protocol 3 **(D)**. The red arrows indicate the time after which HAV was added on sub-confluent monolayers of FRhK-4 cells.

The impedance of mock-infected cells measured depending on the infection protocol used is displayed in Figure 3A and the means of CI values are given in Table 1. When the cells were incubated in FBS-free medium for 2 h, there was a significant drop of CI values from 4.98 ± 0.50 and 5.20 ± 0.52 (CI before infection) to 1.80 ± 0.31 and 2.36 ± 0.39 (CI_min_) for Protocol 1 and Protocol 2 respectively. CI then remained low and stable between 1.73 ± 0.50 (CI at the end of the experiment) and 2.39 ± 0.30 (CI_max_) with the addition of new FBS-free medium (Protocol 1), whereas the addition of medium containing FBS (Protocol 2) induced an increase of CI values as high as 4.94 ± 0.20 (CI_max_). The cells treated under Protocol 3 also presented a biphasic response, with the initial decrease from 5.05 ± 0.52 to 3.29 ± 0.50 (CI_min_) followed by an increase ultimately reaching 5.32 ± 0.13 (CI_max_). Despite very close CI_max_ values, the time to reach this highest CI value was longer with Protocol 2 than with Protocol 3. The CI values remained stable for longer and in the same range for Protocol 2 and Protocol 3 until the end of the experiment.

**Table 1:** Cell Index (CI) values in FRhK-4 cells infected or not with HAV.

**Cell index (CI) (Mean ±s.e.m.)**	**Protocol 1**		**Protocol 2**		**Protocol 3**	
	**Mock**	**HAV**	**Mock**	**HAV**	**Mock**	**HAV**
CI before infection	4.98 ± 0.50	5.27 ± 0.57	5.20 ± 0.52	4.86 ± 0.48	5.05 ± 0.52	4.84 ± 0.44
CI_min_ p.i.	1.80 ± 0.31	1.96 ± 0.38	2.36 ± 0.39	2.07 ± 0.33	3.29 ± 0.50	3.33 ± 0.49
CI_max_ p.i.	2.39 ± 0.30	2.07 ± 0.12	4.94 ± 0.20	3.34 ± 0.45	5.32 ± 0.13	4.59 ± 0.18
CI at end of experiment	1.73 ± 0.50	Around 0	4.42 ± 0.41	Around 0	4.45 ± 0.50	Around 0

*Cell index (CI) values were continuously recorded with the xCELLigence system in mock-infected and in HAV-infected cells. The averages of CI values obtained before challenging FRhK-4 cells with HAV and at the end of experiments (i.e., 7 days post-infection); and the averages of CI values of the minimum and maximum impedance response (i.e., CI_min_ and CI_max_ respectively) following the infection of FRhK-4 with HAV were calculated. The values are expressed as mean CI ± s.e.m. obtained from four experiments performed in duplicate (n = 8)*.

Even if the presence of FBS clearly influenced the CI_min_ and CI_max_ of mock-infected cells, cell impedance remained stable in mock-infected cells for the entire duration of the experimental infection.

**Figure 4:**
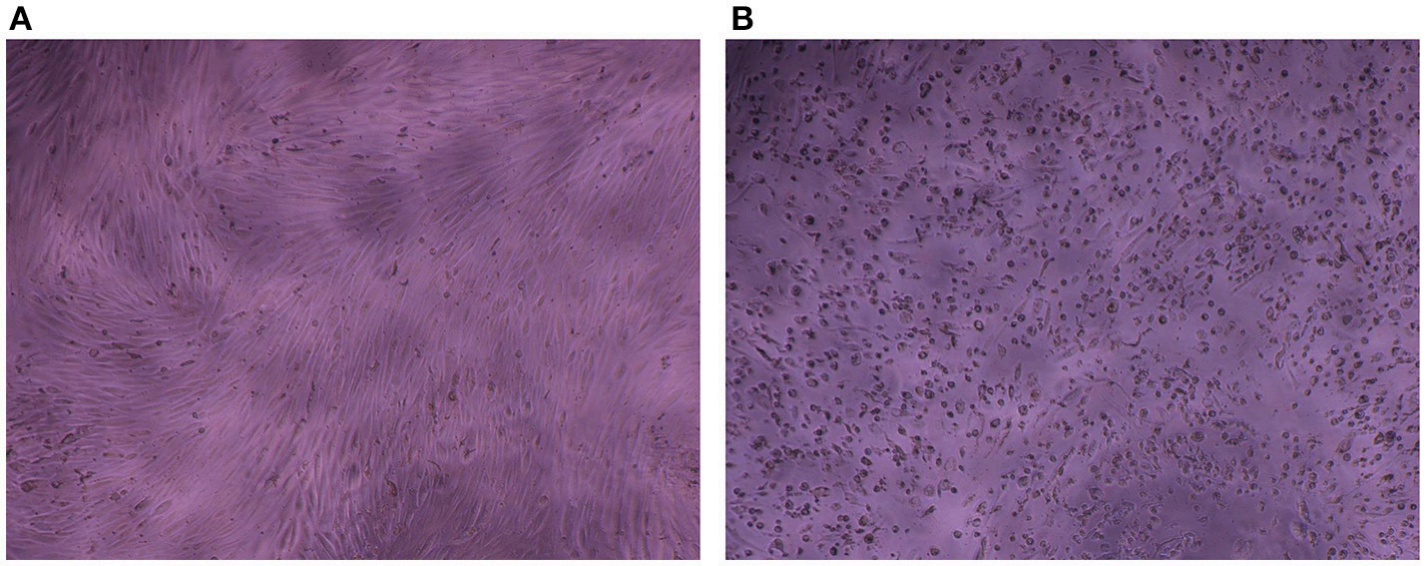
Cytopathic effect induced by HAV in FRhK-4 cells. Phase contrast images of mock-infected **(A)** and HAV-infected cells **(B)** at 7 days p.i.

The impedance of HAV-infected cells measured in real time according to the infection protocol used is displayed in Figure 3B (Protocol 1), Figure 3C (Protocol 2), and Figure 3D (Protocol 3). In the same way as in mock-infected cells, the culture medium used for cell infection (e.g., presence of FBS) affected the extreme values of CI (CI_min_ and CI_max_) in HAV-infected cells. Nevertheless, the kinetic curves of CI values in HAV-infected FRhK-4 cells presented a triphasic response for all the protocols used, and the means of CI values surrounding each phase (CI before infection, CI_min_, CI_max_, and CI at the end of the experiment) are shown in Table 1. The first two phases presented the same profile as in mock-infected cells, the decrease until CI_min_ followed by an increase until CI_max_. Subsequently, unlike mock-infected cells, which maintained stationary CI values, HAV-infected cells displayed a time-dependent decrease of CI down to zero for all three of the protocols applied. As shown in Figure 4, CI of HAV-infected cells reached zero when cells died as a consequence of HAV-induced cytopathic effect (CPE).

As a whole, these results indicate that the kinetic profiles of cell impedance are influenced by the culture medium, whereas they do not appear to differ between mock and HAV-infected cells until the CI_max_ value from which an HAV-induced drop in CI occurs.

### Effect of cell culture medium on the CI_min_ and CI_max_ values in mock–and HAV-infected cells

**Figure 5:**
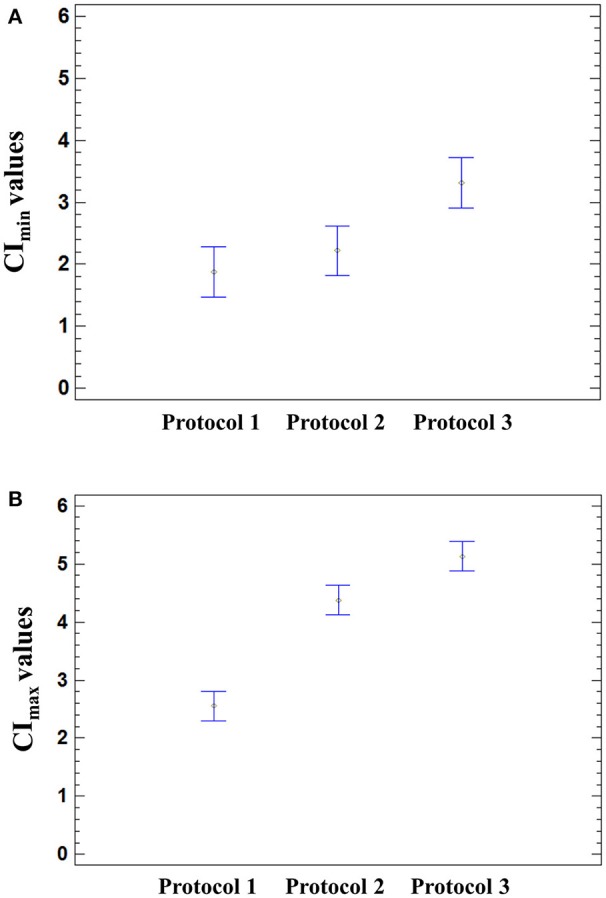
Effect of the infection protocol used on the CI values. Significant effect of the medium used for HAV infection was found on the CI_min_ (*p* = 0.0026) and CI_max_ (*p* < 0.0001) using a one-way ANOVA. Multiple comparison analysis testing displayed population marginal means with standard error of CI_min_ **(A)** and CI_max_ values **(B)**. Two means are significantly different if their intervals are disjoint and are not significantly different if their intervals overlap.

The influence of HAV and culture medium on the CI_min_ and CI_max_ values was further assessed by using a two-way ANOVA, and the results of the multiple comparison tests are displayed in Figure 5.

The means of CI_min_ and CI_max_ values reached in HAV-infected cells were not significantly different from those of mock-infected cells (ANOVA; *p* = 0.9320 for CI_min_ and *p* = 0.1410 for CI_max_). The mean CI_min_ values of cells incubated with Protocol 1 and Protocol 2 were significantly different from the mean CI_min_ value of cells incubated with Protocol 3 (ANOVA; *p* = 0.0026), regardless of the presence of HAV (Figure 5A). In the same way, the cell culture medium significantly influenced the CI_max_ values (ANOVA; *p* < 0.0001) (Figure 5B).

The cell culture medium strongly influenced the CI values, whereas only the drop in CI was HAV-dependent.

### Comparison of the three infection protocols in terms of time-dependent CI decrease

To determine which infection protocol had the earliest drop in CI values, the times taken for 25, 50, and 75% of decrease of the CI_max_, termed respectively TCI_25_, TCI_50_, and TCI_75_, were determined for each protocol (Table 2).

**Table 2:** Characterization of CI decrease in HAV-infected cells according to the infection protocol used.

**Time post-infection (h p.i.)**	**Protocol 1**	**Protocol 2**	**Protocol 3**
TCI_max_	38.87 ± 6.39	46.96 ± 3.70	42.67 ± 5.79
TCI_25_	64.31 ± 4.91	74.27 ± 5.65	71.77 ± 5.02
TCI_50_	83.90 ± 6.12	91.82 ± 6.77	91.78 ± 6.28
TCI_75_	106.50 ± 9.01	113.96 ± 8.94	118.21 ± 8.04

*The times taken to reach the CI_max_ (TCI_max_) in FRhK-4 cells infected with HAV are indicated for each infection protocol. HAV-dependent decrease in CI values occurred from the CI_max_. The times to CI_25_, CI_50_, and CI_75_ were determined as the time point at which the CI values for each cell index curve had decreased respectively by 25, 50 and 75% of the CI_max_. Data are presented as mean Time (hours p.i.) ± s.e.m. obtained from four experiments performed in duplicate (n = 8)*.

Following infection, the CI_max_ values were reached 38.87 ± 6.39 h post-infection (h p.i.) for Protocol 1, 46.96 ± 3.70 h p.i. for Protocol 2 and 42.67 ± 5.79 h p.i. for Protocol 3. The HAV-induced CI decrease occurred slightly earlier with Protocol 1 than with Protocols 2 and 3. Nevertheless, the time ranges did not exceed 12 h p.i. between Protocol 1 and the other two. Statistical analysis confirmed that the protocol used did not significantly influence the times to reach a 50% decrease (CI_50_) (one-way ANOVA; *p* = 0.0924).

These results therefore indicate that none of the three protocols would lead to significant time saving.

### RTCA vs. cell-viability assays for detecting HAV-induced cytopathic effect

The RTCA assay measuring HAV-induced cytopathic effect was further compared to a traditional end-point assay measuring dehydrogenase activity in live cells. Results of viability assays were expressed as the percentage of dead cells occurring over time of infection (48, 65, 72, 78, 88, 96, 120, and 168 h p.i.) as compared to the percentage of CI decrease induced by HAV (Table 3).

**Table 3:** Correlation between RTCA assay and cell viability assay.

**Time (h p.i.)**	**Protocol 1**		**Protocol 2**		**Protocol 3**	
	**Cell mortality (%)**	**CI decrease (% CI_max_)**	**Cell mortality (%)**	**CI decrease (% CI_max_)**	**Cell mortality (%)**	**CI decrease (% CI_max_)**
48	28.06 ± 2.90	7.34 ± 0.84	4.41 ± 2.83	10.71 ± 4.53	1.75 ± 13.37	6.35 ± 1.83
65	19.86 ± 3.92	22.13 ± 2.91	28.80 ± 2.50	14.05 ± 3.58	8.07 ± 7.09	15.35 ± 2.44
72	26.14 ± 4.30	31.86 ± 2.99	30.59 ± 5.89	18.36 ± 4.43	20.89 ± 4.20	22.50 ± 2.86
78	23.95 ± 8.10	39.55 ± 3.02	28.93 ± 2.64	26.73 ± 4.25	23.12 ± 9.27	29.93 ± 3.11
88	43.98 ± 7.93	52.36 ± 3.07	49.01 ± 8.64	41.72 ± 4.17	25.06 ± 2.10	42.17 ± 3.04
96	51.28 ± 2.95	62.01 ± 3.17	48.78 ± 3.02	52.36 ± 4.20	41.22 ± 5.31	51.47 ± 3.03
120	61.97 ± 13.70	83.40 ± 2.73	74.53 ± 3.67	77.22 ± 2.81	68.13 ± 7.25	73.82 ± 2.42
168	94.10 ± 1.97	99.70 ± 0.19	88.71 ± 1.76	97.37 ± 1.04	88.01 ± 1.83	92.79 ± 1.43

*Cell viability assay was carried out using the WST-8 assay in mock-infected and HAV-infected FRhK-4 cells at different times (h) post-infection (p.i.), and data are presented as the mean percentages of cell mortality. At the same time points, CI values were compared to the CI_max_ and data are presented as the mean percentages of CI_max_ decrease occurring in HAV-infected cells. The values are expressed as mean percentages (Cell mortality or CI decrease) ± s.e.m. obtained from three experiments performed in duplicate*.

At 48 h post-infection, a slight increase in cell mortality was only observed with Protocol 1 (28.06 ± 2.90%). In the meantime, the CI_max_ values were reached (between 38.87 ± 6.39 and 46.96 ± 3.70 h p.i.) whichever protocol was used. The mortality of FRhK-4 cells increased with the time of viral infection as the CI values decreased, for all protocols used. Half of the cells died between 88 h p.i. and 96 h p.i. with Protocol 1 and between 96 h p.i. and 120 h p.i. with Protocols 2 and 3. In the same time lapse, a decrease of 52.36 ± 3.07% of the CI_max_ was observed at 88 h p.i. with Protocol 1, and of 52.36 ± 4.20% and 51.47 ± 3.03% with Protocols 2 and 3 respectively at 96 h p.i. Finally, almost complete cell death was observed at 168 h p.i. as the CI drop was 92.79 ± 1.43–99.70 ± 0.19% of the CI_max_.

Taken together, the incidence of cell death increased over time in the HAV-infected cells as determined by decrease of both cell viability and cell index values. The RTCA assay was able to predict the HAV-induced cytopathic effect (CPE) at the same time as the cell viability assay, regardless of the protocol used, but earlier than visual observation by using a light microscope.

### Determination of the sensitivity of the RTCA assay for detecting HAV in FRhK-4 cells according to the viral inoculum level

**Figure 6:**
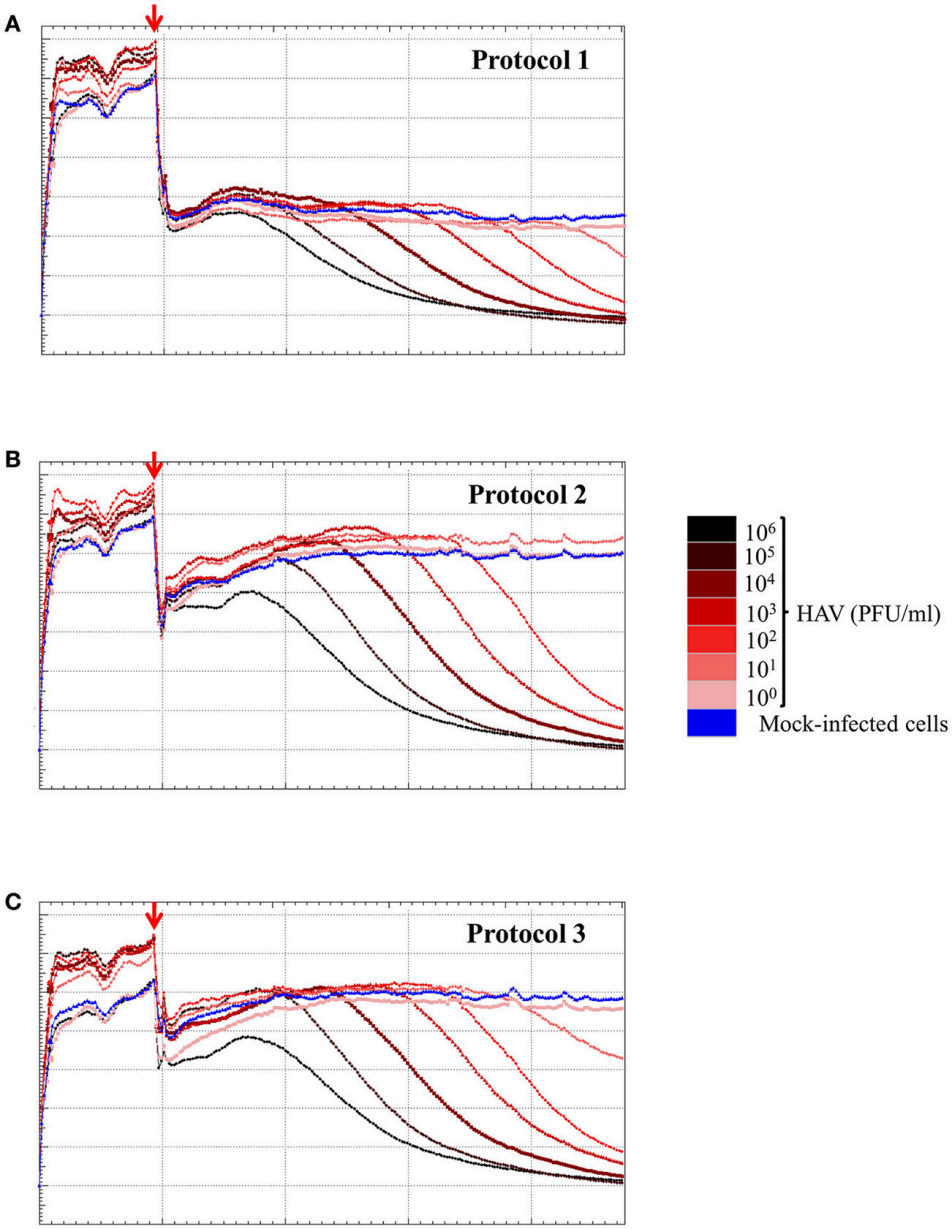
HAV infection induces a dose-dependent effect as measured by the RTCA assay. Kinetic curves of CI values in FRhK-4 cells uninfected (blue) or infected with different amounts of HAV [from 10^5^ PFU/well (black) to 0.1 PFU/well (pink)] using protocol 1 **(A)**, protocol 2 **(B)**, and protocol 3 **(C)**. The red arrows indicate the time after which HAV was added on sub-confluent monolayers of FRhK-4 cells.

To determine the sensitivity of the RCTA assay, FRhK-4 cells were infected with serial ten-fold dilutions of HAV according to the viral protocol, and kinetic measurements of CI values are displayed in Figure 6. The kinetic curves showed that the CI drop in HAV-infected cells was delayed with the dilution of viral inoculum regardless of the protocol used.

The times to reach the CI_50_ values (TCI_50_) are reported in Table 4 for each protocol according to the viral concentration. Kinetic TCI_50_ values increased regularly for every one-log decrease for viral suspensions ranging from 10^5^ to 10^3^ PFU/ml for Protocol 1 and from 10^5^ to 10^1^ for Protocol 2 and Protocol 3. Indeed, the ΔTCI_50_ was between 21.28 and 26.26 for Protocol 1, 19.95 and 22.39 for Protocol 2 and 18.34 and 22.08 for Protocol 3. However, ΔTCI_50_ obtained between the two highest viral concentrations, 10^6^ and 10^5^ PFU/ml, ranged from 7.73 to 8.63 whichever protocol was used. These results indicated a correlation between the HAV inoculum level and the kinetic parameter measuring cytopathogenesis, TCI_50_.

**Table 4:** Kinetic parameters TCI_50_ according to the inoculum level for the three infection protocols.

**[HAV] (PFU/ml)**	**Protocol 1**		**Protocol 2**		**Protocol 3**	
	**TCI_50_ (h)**	**ΔTCI_50_**	**TCI_50_ (h)**	**ΔTCI_50_**	**TCI_50_ (h)**	**ΔTCI_50_**
10^6^	71.97 ± 2.23*(8/8)*	/	75.76 ± 3.13*(8/8)*	/	79.86 ± 3.97*(8/8)*	/
10^5^	79.04 ± 2.39*(8/8)*	8.13	88.27 ± 2.61*(8/8)*	8.63	88.12 ± 3.23*(8/8)*	7.73
10^4^	**98.11** ± **5.05** ***(8/8)***	23.11	110.78 ± 2.36*(8/8)*	22.30	115.79 ± 6.90*(8/8)*	21.26
10^3^	117.31 ± 7.99*(6/8)*	21.28	131.14 ± 3.60*(8/8)*	20.61	128.09 ± 3.63*(8/8)*	18.34
10^2^	138.24 ± 21.83*(6/8)*	26.26	**153.43** ± **2.50** ***(8/8)***	22.39	**149.89** ± **4.64** ***(8/8)***	22.08
10^1^	113.09 ± 37.90*(4/8)*	−13.73	173.60 ± 3.63*(6/8)*	19.95	169.01 ± 1.31*(6/8)*	20.49
10^0^	ND *(0/8)*		ND *(0/8)*		ND *(0/8)*	

*Kinetic parameters TCI_50_, which refers to the time taken for 50% reduction in impedance signal response in HAV-infected cells using Protocols 1, 2, and 3 are calculated for every viral concentration. Results are expressed as means of TCI_50_ (hours p.i.) ± s.e.m. The numbers of replicates with a CI drop are italicized and given in parentheses. LOD_100_ is the lowest concentration of HAV having eight CI drops within an experimentation set. TCI_50_ values corresponding to LOD_100_ are indicated in bold. The ΔTCI_50_ corresponds to the time to reach CI_50_ calculated for every CI curve for both inoculum levels. Means of these differences are expressed in hours (h) p.i. ± s.e.m. obtained from four experiments performed in duplicate (n = 8)*.

As shown in Table 4, the limit of detection (LOD), defined as the lowest concentration of HAV likely to be detected by the RTCA assay, was 10^4^ PFU/ml using Protocol 1 and 10^2^ PFU/ml using Protocols 2 and 3. Thus, the RTCA assay is more sensitive when using either Protocols 2 or 3, showing that cell culture condition used for viral infection influenced the sensitivity of the RTCA assay.

### Standard curve for HAV titration by RTCA assay

To evaluate whether the RTCA assay could be used as a titration method for HAV, TCI_50_ was regressed as a linear function of the log_10_ of the HAV concentration (PFU/ml) according to the infection protocol used.

**Figure 7:**
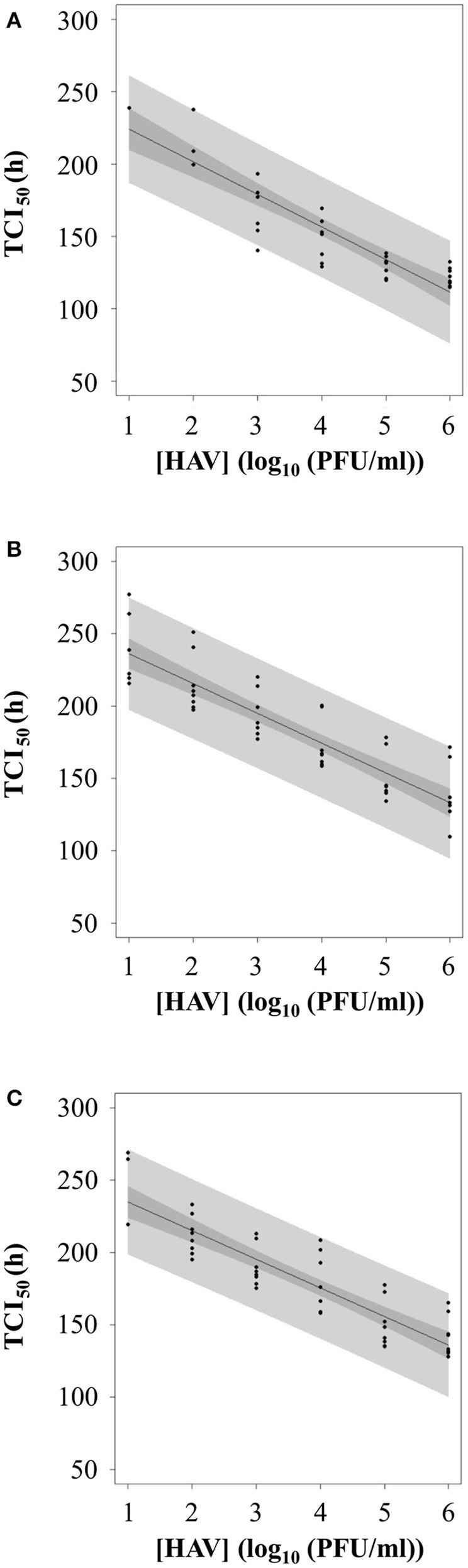
Calibration curve between TCI_50_ and log_10_ of HAV concentration according to the three infection protocols: **(A)** Protocol 1, **(B)** Protocol 2, and **(C)** Protocol 3.

Figure 7 shows the plot of TCI_50_ over log HAV concentration. The relation was linear for Protocols 2 and 3 (Figures 7B,C) with data points scattered above and below the curve for the whole range of concentrations tested. For Protocol 1, the linearity on the whole range of concentration is questionable (Figure 7A), as all TCI_50_ values are above the regression line for 6 log_10_ PFU.

Therefore, the inverse linear relationship established when using Protocols 2 or 3 could be used as standard curves for HAV titration.

## Discussion

The development of methods for the detection of infectious virus is essential to evaluate the virucidal effects of technological treatments applied on foodstuffs for virus removal. In this study, a RTCA-based assay was developed for the detection and quantification of the HM175/18f adapted strain of HAV in FRhK-4 cells.

The cell proliferation study of the FRhK-4 cell line using the xCELLigence system enabled us to identify the optimal density for cell seeding (i.e., 10,000 cells per well) to perform the infection with HAV when a sub-confluence state is achieved 2 days after seeding. Our data also showed that the level of FBS in cell culture medium strongly influenced the impedance kinetic profiles of FRhK-4 cells. Indeed, until the viral-dependent CI drop observed from the CI_max_ values, the sole significant difference observed in CI values of cells infected or not with HAV was linked to the cell culture medium used for viral infection (e.g., effect of the viral infection protocol). The importance of cell culture conditions for having reproducible cell impedance data has been previously underlined by others in the context of screening for drug discovery (Atienzar et al., 2011). Therefore, this technology based on impedance measurement can be a useful tool for the optimization of cell culture conditions (e.g., number of cells at seeding; cell culture medium) which can ensure consistency and reproducibility in the phenotypic characteristics of cells before carrying out a viral infection.

The cytopathic effect (CPE) induced by the adapted HM175/18f strain of HAV in FRhK-4 cells could be recorded in real-time by the RTCA platform and displayed as a virus-specific time-dependent drop in CI values, regardless of the infection conditions used. None of the three infection protocols evaluated significantly influenced the time required to reach a 50% decrease in CI values (TCI_50_). Our data also showed that the decrease in cell impedance of HAV-infected cells was strongly associated with an increase in the incidence of cell death within the infected monolayers. Indeed, a good correlation between the percentages of CI decrease and cell death was found for all three infection protocols. The decrease in the CI values reflected the loss of cell adhesion in HAV-infected cells that underwent cell death. Other studies have reported correlations between cell impedance measurements and cell viability assays based on the activity of mitochondrial reductases when carrying out cytotoxicity assays (Atienzar et al., 2011; Otero-González et al., 2012; Kim et al., 2014).

The RTCA assay developed in this study could be further used as a titration method for HAV since our results showed a linear relation between the HAV inoculum level and the kinetic value of TCI_50_. Other studies revealed that a linear relation can be established between TCI_50_ and a log_10_ concentration of virus (Witkowski et al., 2010; Fang et al., 2011). The linear range covered viral suspensions with concentrations from 10^1^ to 10^6^ PFU/ml when Protocols 2 and 3 were used for HAV infection. The shortest linear range when using Protocol 1 showed that culture conditions influence the quantitative detection of infectious virus by RTCA assay. In the same manner, the impact of cell culture medium on virus-induced CPE kinetics recorded with the RTCA system has recently been shown, which underlines its usefulness for optimizing the efficiency of viral infection (Ramis et al., 2013; Charretier et al., 2018). A similar linear range of 5 log_10_ infectious virus was reported for titration of other viruses such as West Nile virus (WNV) (Fang et al., 2011), infectious bursal virus (IBDV) (Ebersohn et al., 2014), and vaccine strains of dengue virus [chimeric yellow fever dengue (CYD)] (Charretier et al., 2018).

In this study, the RTCA-based assay developed for the titration of infectious particles of HAV (HM175/18f adapted strain) provides several advantages over the gold standard methods currently used for HAV titration, which are based on plaque assays or end-point dilution (TCID_50_) assays (Barrett et al., 1996). Inversely, with the end-point standard assay, the RTCA assay allows for label-free and dynamic monitoring of HAV-induced CPE using impedance as readout. The HAV-induced drop in CI values appeared before it became possible to view cytopathic effect under a light microscope. Thus, this new titration method makes viral diagnosis considerably cheaper and faster, as it provides cell-impedance measurements for the entire duration of viral infection. Traditional plaque assays used for HAV titration take about 13 days whatever the inoculum viral load, whereas the real-time xCELLigence system requires no more than 6 days to cover the 5 log_10_ linear range. It takes 3 days for viral suspensions higher than 10^3^ PFU/ml and 6 days for viral suspensions higher than 10 PFU/ml.

In addition, the viral titer determined by RTCA assay is more accurate since there is no longer the laborious task of manual examination of visible plaques with subjective observation by highly-trained technicians. Regarding the reduction in the overall workload and the consumption of reagents (i.e., e-plate 16 or 96-wells with the high throughput MP xCELLigence system) instead of 6-well plates), the RTCA assay is less expensive than the traditional plaque assay.

The innovative RTCA assay developed in this study for quantitation of the adapted strain of HAV could be implemented to assess the virucidal efficacy of Food-Processing Technologies used in the food industry to control risks associated with HAV-contaminated foods and thus to provide a safer evaluation of risk. The 5 log_10_ linearity range of our RTCA assay would easily validate the efficiency of treatments, since a 4-log_10_ reduction in viral titers is the current criteria for validating an inactivation process (AFSSA, 2009). Methods used for virus extraction from food require adaptations so that samples can be suitable for any cell culture-based detection methods. Indeed, the samples have to be free of any bacterial and fungal contaminations to be assessed by the RTCA assay. Nevertheless, traditional plaque or TCID50 assays are already used to assess the efficacy of treatments (heat, ozone, chlorine, sanitizer…) for virus removal (cultivable viruses such as murine norovirus and adapted strain of HAV) from artificially contaminated food (berries, oysters, leafy vegetables…) (Deboosere et al., 2010; Bozkurt et al., 2015; Araud et al., 2016; Zhou et al., 2017; Brié et al., 2018). In order to avoid microbial contaminations of cell culture, we and others carry out sterilization of extracted samples by filtering through a 0.22-μm-pore-size membrane and add antibiotic and antifungal agents to cell culture medium. Therefore the RTCA assay can easily replace the traditional assays due to its many advantages such as its high-throughput capacity by using the currently available high-throughput MP system.

Regarding the long-term goal which is the detection of HAV from food suspected to be contaminated, many steps remain. The use of cell impedance as readout of virus infectivity should be further evaluated for the detection of the fastidious cultivable wild-type strains of HAV. Indeed, they replicate relatively slowly in cell culture without a CPE. Nevertheless, it has been recently shown that some strains of African swine fever virus could induce significant changes in CI values even for strains lacking a visible CPE in the infected culture (Burmakina et al., 2018). In addition, to overcome the absence of specificity of the RTCA assay, as all cell culture-based methods, it will be necessary to combine its advantage in detecting infectious virus with the specificity of a molecular-based method (RT-qPCR).

In conclusion, the RTCA assay provides a powerful and reliable tool for effective risk management strategies in food industries. It also provides an easy and reliable cell-based screening tool for the process of developing new therapeutic methods, since it can be used to screen several compounds with anti-HAV effects. As a whole, this study contributes to improving risk management in public health issues.

## Author contributions

SL, AF, and SM-L performed experiments. SM-L and SL analyzed data. SM-L, LG statistical study. All authors conceived and designed experiments, wrote the paper and approved the final manuscript.

### Conflict of interest statement

The authors declare that the research was conducted in the absence of any commercial or financial relationships that could be construed as a potential conflict of interest.
